# Long-Term Exposure to Ambient Air Pollution and Myocardial Infarction: A Systematic Review and Meta-Analysis

**DOI:** 10.3389/fmed.2021.616355

**Published:** 2021-03-17

**Authors:** Li Zou, Qiao Zong, Wenning Fu, Zeyu Zhang, Hongbin Xu, Shijiao Yan, Jin Mao, Yan Zhang, Shiyi Cao, Chuanzhu Lv

**Affiliations:** ^1^Department of Neurology, Renmin Hospital of Wuhan University, Wuhan, China; ^2^Department of Neurology, Taihe Hospital, Hubei University of Medicine, Shiyan, China; ^3^School of Public Health, Tongji Medical College, Huazhong University of Science and Technology, Wuhan, China; ^4^School of Nursing, Tongji Medical College, Huazhong University of Science and Technology, Wuhan, China; ^5^Key Laboratory of Emergency and Trauma, Ministry of Education, College of Emergency and Trauma, Hainan Medical University, Haikou, China; ^6^School of International Education, Hainan Medical University, Haikou, China; ^7^School of Medicine and Health Management, Tongji Medical College, Huazhong University of Science and Technology, Wuhan, China; ^8^Department of Emergency, Hainan Clinical Research Center for Acute and Critical Diseases, The Second Affiliated Hospital of Hainan Medical University, Haikou, China; ^9^Emergency and Trauma College, Hainan Medical University, Haikou, China; ^10^Research Unit of Island Emergency Medicine, Medical University, Chinese Academy of Medical Sciences, Haikou, China

**Keywords:** air pollution, particulate matter, myocardial infarction, meta-analysis, PM2.5 (AQI), PM10

## Abstract

**Background and Objective:** An increasing number of epidemiological original studies suggested that long-term exposure to particulate matter (PM_2._5 and PM_10_) could be associated with the risk of myocardial infarction (MI), but the results were inconsistent. We aimed to synthesized available cohort studies to identify the association between ambient air pollution (PM_2.5_ and PM_10_) and MI risk by a meta-analysis.

**Methods:** PubMed and Embase were searched through September 2019 to identify studies that met predetermined inclusion criteria. Reference lists from retrieved articles were also reviewed. A random-effects model was used to calculate the pooled relative risk (RR) and 95% confidence intervals (CI).

**Results:** Twenty-seven cohort studies involving 6,764,987 participants and 94,540 patients with MI were included in this systematic review. The pooled results showed that higher levels of ambient air pollution (PM_2.5_ and PM_10_) exposure were significantly associated with the risk of MI. The pooled relative risk (RR) for each 10-μg/m^3^ increment in PM_2.5_ and PM_10_ were 1.18 (95% CI: 1.11–1.26), and 1.03 (95% CI: 1.00–1.05), respectively. Exclusion of any single study did not materially alter the combined risk estimate.

**Conclusions:** Integrated evidence from cohort studies supports the hypothesis that long-term exposure to PM_2.5_ and PM_10_ is a risk factor for MI.

## Introduction

The incidence and prevalence of cardiovascular diseases have increased in recent decades, and cardiovascular disease has become one of the main causes of death among adults (1–3). Myocardial infarction (MI) is an acute and severe cardiovascular disease that generally can endanger the life of patients and has become a serious public health problem (4). The causes of MI are complex and are related to lifestyle, diet structure, genetic factors, and environmental factors, including air pollution (5–7). Studies have shown that reducing modifiable risk factors may contribute to the prevention and control of MI (8–10), which is of considerable public health importance.

Air pollution, especially particulate matter (PM), has been increasingly investigated as an environmental risk factor for MI morbidity and mortality recently. However, studies have had modest sample sizes and reported inconclusive results (11–13). These inconsistent and controversial results indicate the need to quantitatively synthesize and interpret the available evidence to provide more explicit information for policy decisions and clinical use. Meta-analysis is a statistical tool that can be used to integrate results of multiple independent studies considered to be combinable for a more precise estimation (14, 15). Although previous meta-analyses have examined associations between air pollution exposure and MI, their studies have mostly included cross-sectional literatures (13), and much new research has been recently published. Therefore, more meta-analyses with cohort studies are urgently needed.

Taking into consideration the inconsistent conclusions and the limitations of existing epidemiological studies and the flaws of previous meta-analyses, we therefore performed a systematic review and meta-analysis of cohort epidemiological studies to examine the potential associations between air pollution exposure and the risk of MI. Given the heavy economic and health burden of curing MI, the results of our study may provide additional practical and valuable clues for the prevention of MI.

## Methods and Materials

Ethical approval is not required for this systematic review.

### Literature Search Strategy

We conducted this meta-analysis in accordance with the Preferred Reporting Items for Systematic Reviews and Meta-Analysis (PRISMA) (16) and the checklist of items in the Meta-Analysis of Observational Studies in Epidemiology (MOOSE) (17). A systematic literature search of PubMed and Embase was conducted through September 2019 by using the following search terms with no restrictions: “air pollution” or “particulate matter” or “air pollutants” or “PM_10_”or “PM_2.5_” or “air quality” in combination with “myocardial infarction” or “heart attack” or “acute coronary syndrome” or “cardiovascular disease” or “heart disease.” The language was restricted to English. Additionally, reference lists of the retrieved original articles and relevant review articles were also scrutinized to identify further pertinent studies.

### Study Selection

Studies meeting the following criteria were included in the meta-analysis: (1) the study design was cohort; (2) the exposure of interest was ambient air pollution; the endpoint of interest was the incidence of MI; and (3) the relative risk (RR) and the corresponding 95% confidence interval (CI) of MI relating to ambient air pollution were reported or could be calculated from the data provided. Animal studies, clinical trials, reviews, letters, and commentaries were excluded. Only studies with detailed information on both ambient air pollution and the incidence of MI was included.

### Data Extraction and Quality Assessment

Two investigators (WF and SC) extracted the following information from the studies: first author, publication year, country, study period, age, number of cases, size of cohort, and time windows of exposure. Discrepancies were resolved by discussion with a third investigator (SC).

The Newcastle-Ottawa Scale, used to evaluate the qualities of cohort studies (18), is a nine-point scale allocating points based on the selection of participants, comparability of groups, and exposure/outcome. This scale awards a maximum of nine points to each study: four for selection of participants and measurement of exposure, two for comparability of cohorts or cases and controls on the basis of the design or analysis, and three for assessment of outcomes and adequacy of follow-up. Studies scoring 0–3 points, 4–6 points, and 7–9 points were categorized as low, moderate, and high quality of studies, respectively. When studies had several adjustment models, we extracted those that reflected the maximum extent of adjustment for potentially confounding variables. Each study was rated independently by two authors (WF and JM). Discrepancies were resolved by discussion with a third investigator (SC).

### Statistical Analyses

We used RR to measure the association between ambient air pollution and the risk of MI and the random effects model was used to calculate an overall pooled RR for the main analysis.

The *Q* statistic with a significance level at *P* < 0.10 and *I*^2^ statistic were used to test heterogeneity. The *I*^2^ statistic measures the percentage of total variation across studies due to heterogeneity rather than chance. It was calculated according to the formula by Higgins (19). We used *I*^2^ to quantify the heterogeneity, with 25%, 50%, and 75% indicating low, moderate and high degrees of heterogeneity, respectively.

In our meta-analysis the following formula was used to calculate the standardized risk estimates for each study:

$$\begin{array}{l} {\text{RR}}_{\left(\text{standardized}\right)}={\text{RR}}_{\text{ (original)}}^{\text{Increment}(\text{10})\text{/Increment}(\text{original})} \end{array}$$

Subgroup analyses were conducted to determine the possible influence of some factors such as state and publication years. We conducted a sensitivity analysis to explore potential sources of heterogeneity and to investigate the influence of various exclusion criteria on the pooled risk estimate. Begg's rank correlation and Egger's linear regression tests were used to assess the potential publication bias (20, 21). Using Duval and Tweedie's non-parametric trim-and-fill method to adjust potential publication bias (22). All analyses were performed using STATA statistical software (version 12.0; College Station, TX, USA), and all tests were two-sided with a significance level of 0.05.

## Results

### Literature Search

**Figure 1:**
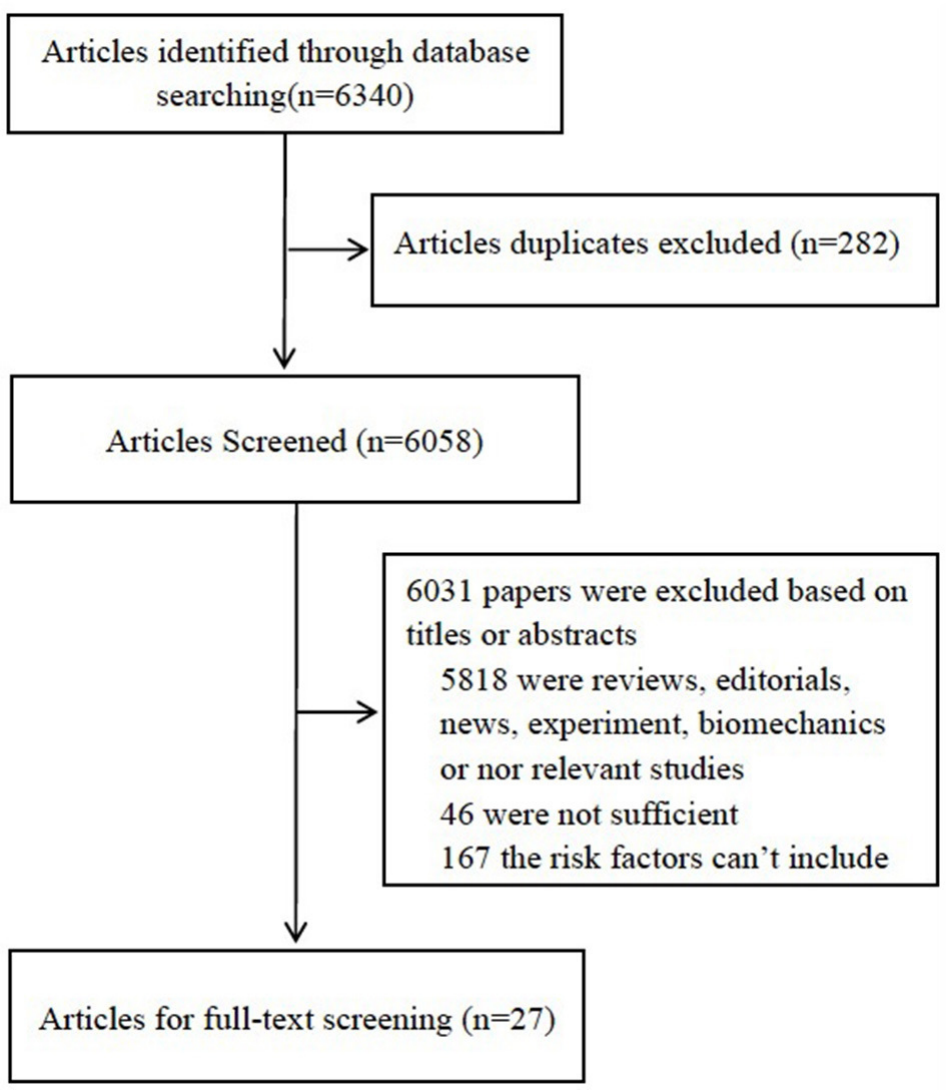
Flow diagram of identification of relevant observational studies of ambient air pollution in relation to the risk of MI.

Figure 1 shows the process of study identification and inclusion. Initially 3,097 and 3,243 citations were retrieved from PubMed database and Embase, respectively. After the exclusion of 282 duplicates, 6,058 potentially relevant studies from electronic databases were identified. Of these, we excluded 6,031 papers because they were experimental, biomechanics, reviews, or irrelevant studies. After full-text review of the remaining 213 articles, 46 articles were excluded because of insufficient data to calculate the risk estimates, and 167 were excluded because they were not a risk factor. Finally, 27 studies (23–49) were included.

### Characteristics of the Studies Included

The main characteristics of the 27 studies on MI and long-term PM exposure in our meta-analysis were summarized in Table 1. These studies were published between 2004 and 2019. Among them, eight studies were from Europe, two studies were from Asia, and twelve studies were from America. The size of the cohorts ranged from 1,120 to 4,404,046 for a total of 6,764,987 subjects. The exposure measure was PM_2.5_ in seventeen studies and PM_10_ in eleven studies; six publications investigated the association of MI with exposure to both PM_2.5_ and PM_10_.The end point was MI incidence (sixteen studies), MI mortality (twelve studies), and only one study MI hospital, and two studies included MI incidence and mortality. Twenty studies were published after 2010, and seven studies before 2010. The quality assessment scores ranged from 6 to 9, with an average score of seven points, representing satisfactory quality of the studies.

**Table 1:** Main characteristics of the studies included involving ambient air pollution and the risk of myocardial infarction.

**Author**	**Year**	**Country (state)**	**Exposure**	**Study period**	**Age**	**Sample size**	**Gender, female (%)**	**Time windows of exposure**	**Endpoints**	**No. of cases**
Jaana Hartiala	2016	American (North America)	PM_2.5_	1998–2010	≥64	6,575	32	2001–2007	Incidence	288
Laura A	2016	American (North America)	PM_2.5_	2001–2011	20–93	9,334	39	2002–2009	Incidence	704
Malik A	2019	American (North America)	PM_2.5_	2003–2008	60.6 ± 12.2	5,650	25	2005–2008	Mortality	978
Hyeanji Kim	2017	Korea (Asia)	PM_2.5_	NA	≥18	136,094	50.9	2007–2013	Incidence	1,881
Krishnan B	2016	England (Asia)	PM_10_	2003–2006	NA	79,288	NA	2003–2006	Incidence	NA
Teresa To	2015	Canada (North America)	PM_2.5_	NA	40–59	29,549	100	1980–2006	Incidence	1,233
Giulia C	2014	Sweden (Europe)	PM2.5 PM10	1980– 2006	NA	100,166	NA	1980– 2006	Incidence	5,157
Silvia Koton	2013	Israel (Asia)	PM_2.5_	1995–2011	≤65	1,120	18.8	2005–2011	Incidence	432
Laura A	2017	American (North America)	PM_2.5_	2001–2010	60.8 ± 12.1	5,679	39	2003–2009	Incidence	704
Vicki M	2013	Israel (Asia)	PM_2.5_	1992–1993	≤65	1,120	16.3	2002–2013	Mortality	848
Michael J	2011	American (North America)	PM_2.5_	1996–2005	≥20	124,614	NA	1999–2000	Incidence	722
Daniela N	2011	Italy (Europe)	PM_10_	2002–2005	72.8 ± 13.0	11,450	49	2002–1005	Incidence	950
Kristin A	2007	American (North America)	PM_2.5_	1994–2000	50–79	65,893	100	1998–2000	Incidence	584
Richard W	2012	London (Europe)	PM_10_	2003–2007	40–89	836,557		2003–2006 12 months.	Incidence	13,965
George S	2018	Netherlands (Europe)	PM_2.5_ PM_10_	1993–2010	20–65	23,100	77	2005–2010	Incidence	797
Antonella Zanobetti	2007	American (North America)	PM_10_	1985–1999	≥65	196,131	49.6	1 year	Incidence	22,552
Robin C	2009	South Carolina (North America)	PM_10_	1992–2002	62.4 ± 7.6	66,250	100	48 months	Incidence Mortality	854
Robin C	2008	South Carolina (North America)	PM_10_	1992–2002	30–55	121,700	100	4years	Incidence mortality	2,6,96
Harris He' ritier	2018	Switzerland (Europe)	PM_2.5_	2000–2008	>30	4,404,046	52	2003–2008	Mortality	19,261
Hong Chen	2016	Canada (North America)	PM_2.5_	1999–2011	>35	8,873	35	2001–2011	Mortality	4,016
C. Arden Pope	2004	Canada	PM_2.5_	1982–1988	≥30	319,000 to 500,000	NA	1984 1986 1988	Mortality	47.00%
Rob Beelen	2014	Netherlands (Europe)	PM_2.5_ PM_10_	1992–1996	46.0 (10.2)	22,136			Mortality	117
R Beelen	2009	Netherlands (Europe)	PM_2.5_	1987–2000	55–69	120,852	30.9	1996–2000	Mortality	4,243
Cathryn Tonnea	2015	London (Europe)	PM_2.5_ PM_10_	2003–2007	≥25	18,138	32		Mortality	390
Robin C	2011	South Carolina (North America)	PM_2.5_ PM_10_	1986–2003	40–75	17,545	0	24 36 46 months	Mortality	646
Stephanie von Klot	2005	Finland (Europe)	PM_10_	1992–2001	≥35	22,006	NA	1995–1999	Hospital	2,321
Anke Huss	2010	Switzerland (Europe)	PM_10_	1990–2005	>30	12,122	NA	2000–2005	Mortality	8,192

### Results of Meta-Analysis

We employed meta-analysis to assess the association of PM_2.5_ and PM_10_ with MI.

#### Association Between PM_2.5_ and the Risk of MI

**Figure 2:**
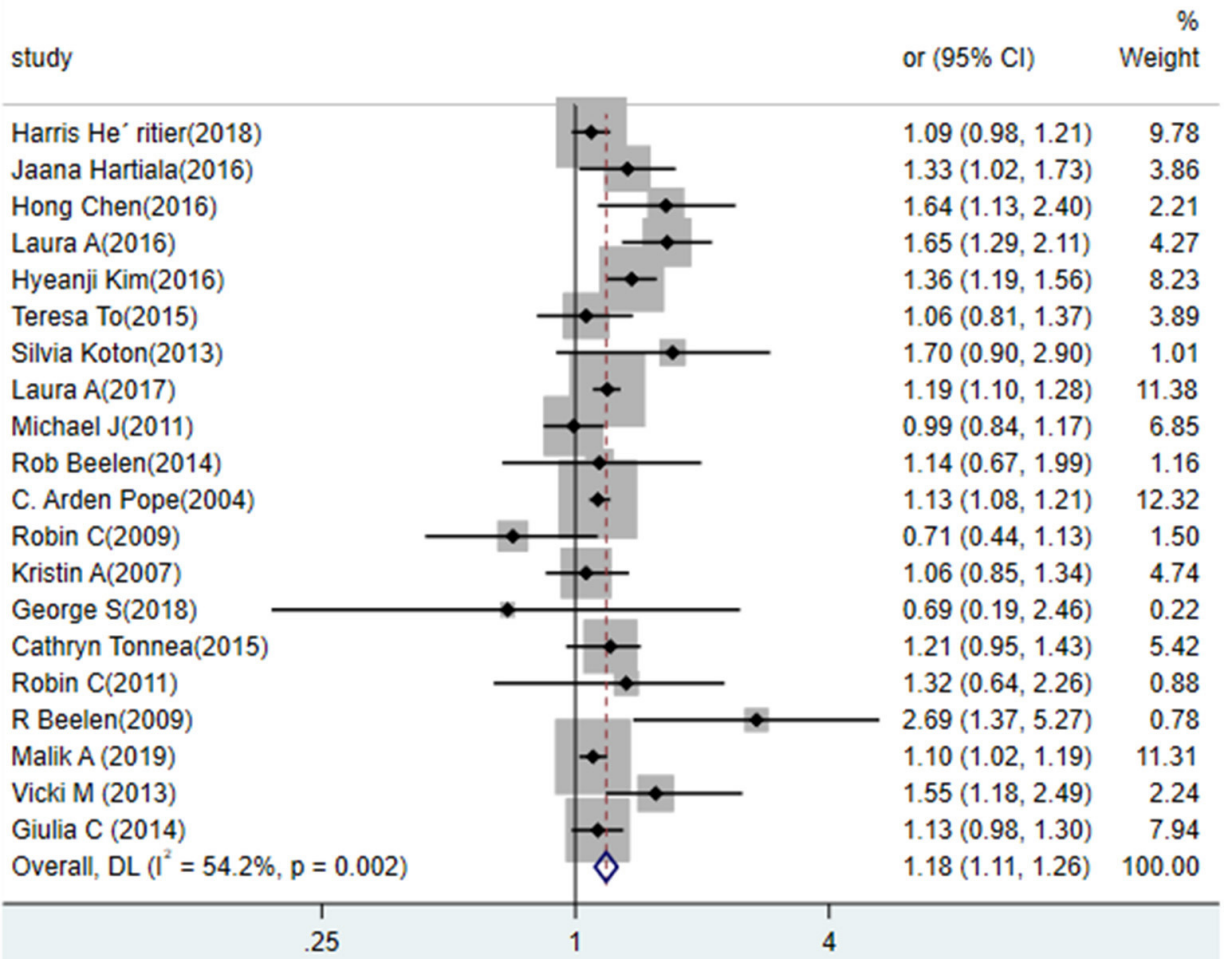
Association between exposure to PM_2.5_ and the risk of MI in a meta-analysis of cohort studies. Weights are from random- efforts model.

The results from random-effects meta-analysis of ambient air pollution (PM_2.5_ and PM_10_) and the risk of MI were shown in Figure 2. Twenty studies reported the effect of PM_2.5_ exposure on the risk of MI and the pooled estimates suggested a positive relationship. Results from standardized data showed that a 10 mg/m^3^ increase in PM_2.5_ exposure was positively associated with the risk of MI (RR = 1.18, 95% CI: 1.11 to 1.26), and there was a moderate heterogeneity (*p* = 0.002; *I*^2^ = 54.2%). Subgroup analyses by state, and publication year (before 2010 vs. after 2010) showed no statistically significant difference in results (Table 2). Every single pooled result of subgroup showed the positive and statistically significant relationship between exposure to PM_2.5_ and the risk of MI.

**Table 2:** Results of subgroup analyses about ambient air pollution and the risk of myocardial infarction.

**Subgroup**	**PM2.5**					**PM10**				
	**Number of studies**	**RR**	**95% CI**	***P*** **for heterogeneity**	***I*****-square (%)**	**Number of studies**	**RR**	**95% CI**	***P*** **for heterogeneity**	**I-square (%)**
**State**										
North America	11	1.15	1.08–1.22	0.014	55.0	2	1.06	0.97–1.15	0.001	85.2
Europe	6	1.12	1.03–1.22	0.017	61.3	5	1.02	0.99–1.05	0.026	67.6.
Asia	3	1.39	1.23–1.58	0.644	48.8	1	—	—	—	—
**Publication years**										
Before 2010	4	1.12	0.87–1.44	0.016	71.1	3	1.06	0.99–1.13	0.187	37.5
After 2010	13	1.16	1.09–1.24	0.000	66.6	11	1.02	1.00–1.05	0.041	47.2
**Populations**										
General	9	1.19	1.06–1.33	0.021	55.5	6	1.03	0.99–1.07	0.281	20.2
Specific	10	1.19	1.10–1/30	0.009	57.5	8	1.04	0.99–1.08	0.031	54.7

#### Association Between PM_10_ and the Risk of MI

**Figure 3:**
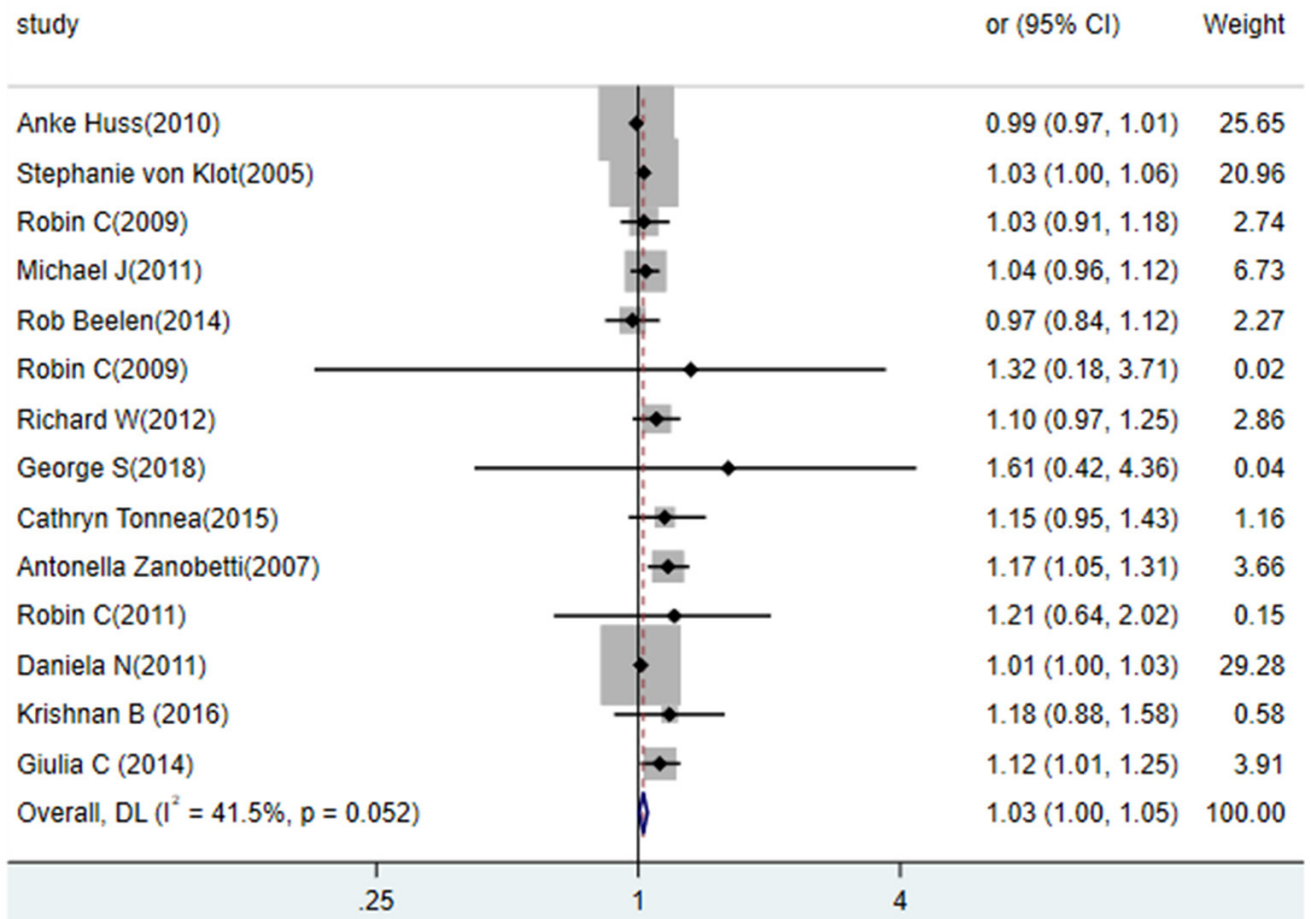
Association between exposure to PM_10_ and the risk of MI in a meta-analysis of cohort studies. Weights are from random- efforts model.

Fourteen studies investigated the association of PM_10_ exposure with the risk of MI (Figure 3). The pooled estimates of these studies indicated that a 10 mg/m^3^ increase in PM_10_ was associated with a higher risk of MI (RR = 1.03, 95% CI: 1.00 to 1.05), and the heterogeneity is (*p* = 0.052, *I*^2^ = 41.5%). We conducted subgroup analyses by state, and publication year (before 2010 vs. after 2010). In general, these subgroup analyses showed no statistically significant difference in results.

### Sensitivity

Sensitivity analyses was conducted to find potential sources of heterogeneity in the association between air pollution (PM_2.5_ and PM_10_) and MI risk, to examine the influence of various exclusions on the combined RR and assess the robustness of all results. The pooled RR did not materially change, for PM_2.5_ and PM_10_, the both overall combined RR did not materially change, with a range from 1.17 (95% CI: 1.10–1.24) to 1.19 (95% CI: 1.11–1.26), and 1.02 (95% CI: 1.00–1.04) to 1.03 (95% CI: 1.01–1.05), respectively.

### Publication Bias

**Figure 4:**
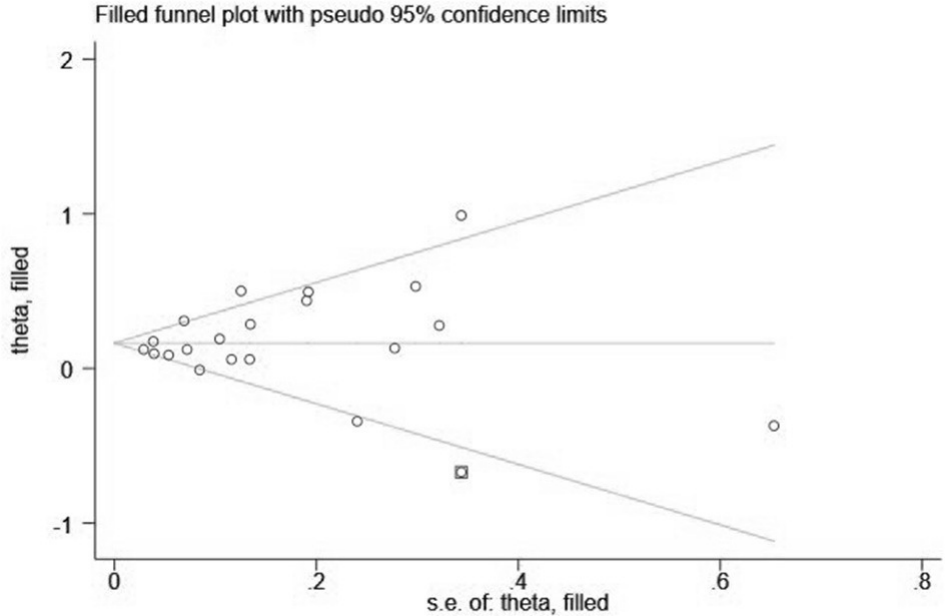
Funnel plot with 95% confidence limits of PM_2.5_ and the risk of MI.

**Figure 5:**
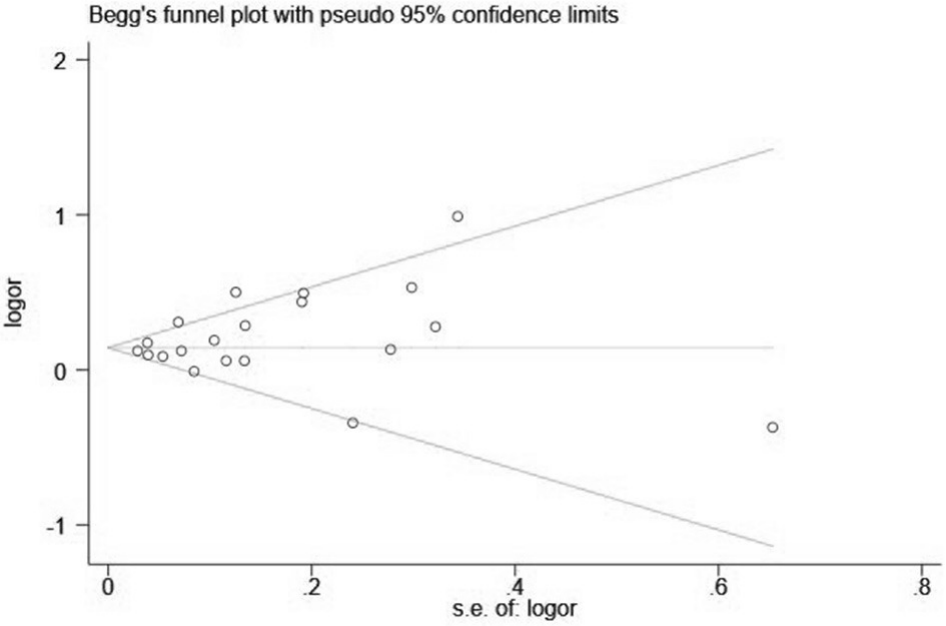
Filled funnel plot of *RR* from studies that investigated the association between PM_2.5_ and the risk of MI.

**Figure 6:**
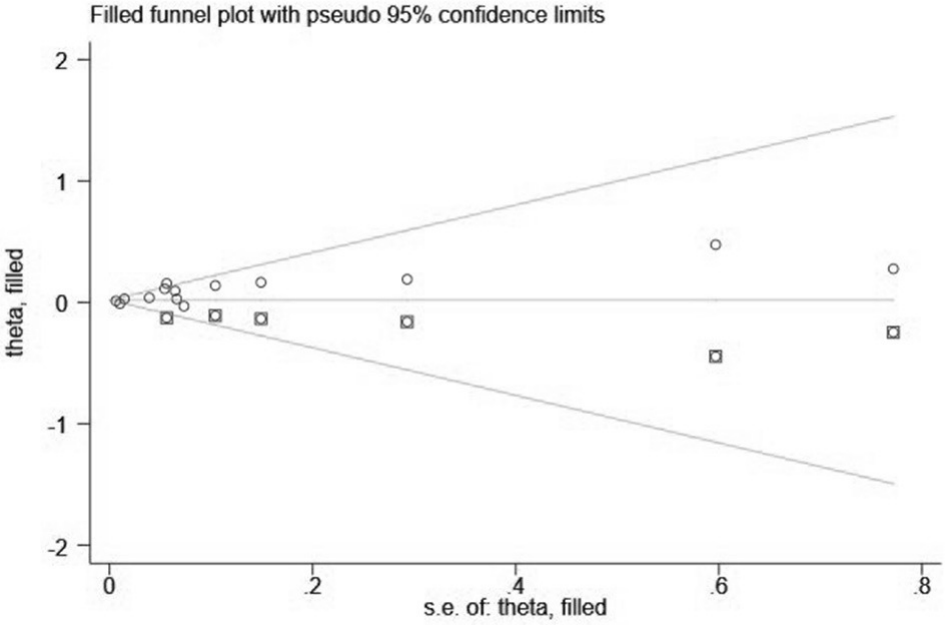
Funnel plot with 95% confidence limits of PM_10_ and the risk of MI.

**Figure 7:**
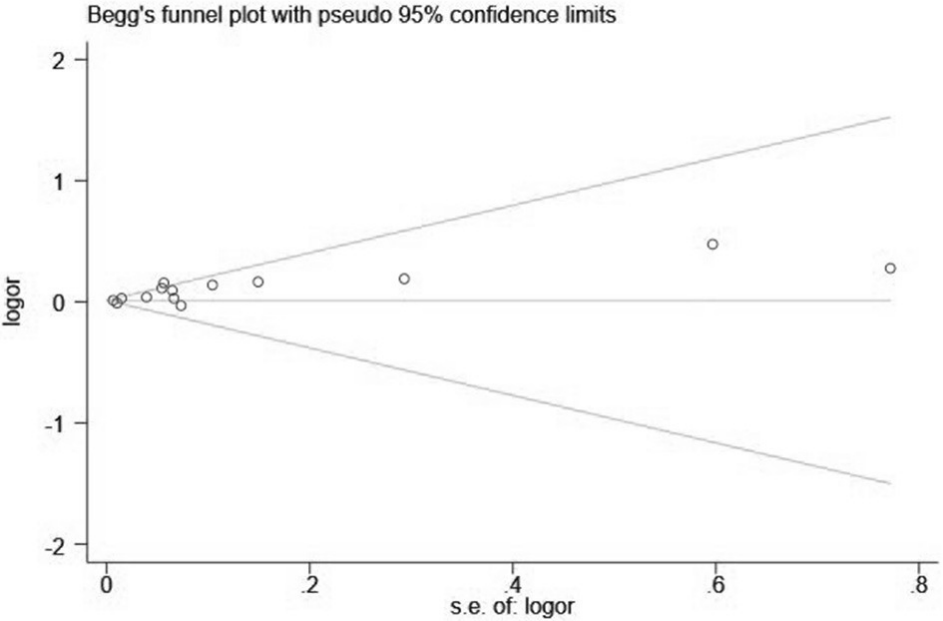
Filled funnel plot of *RR* from studies that investigated the association between PM_10_ and the risk of MI.

Visual inspection of the funnel plot showed significant asymmetry (PM_2.5_: Figures 4, 5; PM_10_: Figures 6, 7). For PM_2.5_ and PM_10_, the Egger test indicated publication bias, but the Begg test did not (PM_2.5_: Egger, *Z* = 5.385, *p* = 0.000, Begg, *t* = 1.44: *p* = 0.168; PM10: Egger, *Z* = 2.283 *p* = 0.022, Begg, *t* = 1.22 *p* = 0.830). We used the trim-and-fill method to evaluate the impact of any potential publication bias, and the results showed that one and five potentially missing studies would be needed to obtain funnel plot symmetry for the association of MI and air pollution (PM_2.5_, PM_10_), respectively. After using the trim-and-fill method, both the corrected *RR* for PM _2.5_ was 1.18 (95% *CI*: 1.10 to 1.26; random-effects model, *p* = 0.000), for PM_10_ was 1.03 (95% CI: 1.00 to 1.06; random-effects model, *p* = 0.027), which suggested the both pooled RRs were not substantially changed by the correction for potential publication bias.

## Discussion

Myocardial infarction results in a high medical burden for families and society and remains a worldwide public health challenge (50). Ascertaining the risk factors of MI could provide significant information for the prevention of MI. Our meta-analysis has quantitatively examined the association between long-term exposure to ambient air pollution (PM_2.5_ and PM_10_) and MI. The pooled analysis, which included 27 cohort studies with more than 6.5 million people, showed an inverse association between exposures to air pollutants (PM_2.5_ and PM_10_) and the risk of MI, which was consistent with previous reviews and meta-analyses (13). However, previous meta-analyses included only studies published before 2014, while more recent studies were not included that might report lower estimates or negative results. Additionally, our meta-analysis included all cohorts from studies with reliable data, so that the effect of exposure can be fully and directly analyzed and conclusions are relatively stable.

The mechanisms by which air pollution exposure could contribute to the development of MI might include inflammation, induction of autophagy, and down-regulation of membrane repair protein MG53. Researchers found that inflammation plays an important role in the formation of coronary atherosclerosis and aggravation of plaque instability, and air pollutants can also promote MI by promoting inflammation (51). The second potential mechanism is the induction of autophagy. Several observational studies have shown that autophagy is a normal process for cells to achieve their own metabolism and organelle renewal. Autophagy can maintain the body's metabolism to reduce damage and protect the organism. However, excessive autophagy can lead to apoptosis of cardiomyocytes and aggravate the damage of ischemic-related sites (52). Studies have found that autophagic levels for exposure to air pollutants are significantly higher than in the control group, while the corresponding protein expression levels, MI size decreases, and myocardial cell damage decrease in Farnesoid X receptor (FXR) knock out SD rats. Therefore, it is speculated that exposure to air pollutants promotes the development of MI through FXR-induced autophagy (53). The third possible mechanism is down-regulation of membrane repair protein MG53. Exposure to air pollutants can affect membrane repair through down-regulation of the expression of MG53 protein and aggravation of the severity of ischemia and hypoxia in MI (54).

We also found that long-term exposure to PM_2.5_ has a more pronounced effect than PM_10_ on MI risk in each 10 μg/m^3^ increase, which is in line with previous related research (13). Compared with PM_10_, PM_2.5_ can remain suspended for a longer time in the air and be inhaled into the respiratory tract and directly into the pulmonary alveoli. In addition, PM_2.5_ has a larger superficial area and hence absorbs more chemical constituents than PM_10_. Therefore, PM_2.5_ is probably more harmful on human health than PM_10_ (55, 56).

Considering people's different diets and lifestyles and the prevalence of MI in different regions, we also conducted subgroup analysis by region, and statistically significant differences across subgroups were found except for PM_10_. In Asia, a 10 mg/m^3^ increase in PM_2.5_ exposure was positively associated with the risk of MI (RR = 1.38), which was inconsistent with previous studies. The possible reasons for the differences include inconsistency of study designs and potentially selective reporting of the results for pollutants. However, it is suggested that the Asia region should pay attention to the relationship between air pollution and MI, and more work on air pollution and epidemiology remains to be done. We conducted a subgroup analysis by publication year but found the pooled result of studies before 2010 was not significantly different from that after 2010.

There are several strengths in this meta-analysis. First, all the studies in our analysis were cohort studies, which is considered as stronger measure for demonstrating causation and identification of risk factors than other observational study designs (57). Second, in this comprehensive literature review, we pooled data from 22 cohort studies from several geographical regions in one meta-analysis, thus increasing the statistical power and allowing an investigation of regional patterns. Third, sensitivity analysis and consistent results from various subgroup analyses indicated that our findings were reliable and robust, although heterogeneity existed among the included studies. Furthermore, all the studies were published in the past decade, indicating that data on both the exposure and the outcome are recent and relevant.

Some limitations in the present meta-analysis should be of concern. First of all, the heterogeneity of the studies included was significant and existed through the whole analysis. But we explored the potential heterogeneity resources by subgroup analysis and sensitivity analysis. Second, there are relatively few studies on the mortality and hospitalization of MI, and there is a lack of data required for meta-analysis, so subgroup analysis is not conducted. Third, other air pollutants may have interaction with PM_2.5_ and PM_10_. A compounding effect of all these air pollutants should be examined and quantified on increasing the risk of MI.

### Conclusion

In conclusion, our meta-analysis identified long-term exposure to PM_2.5_ and PM_10_ as a significant risk factors of MI. In light of the heavy economic burden of MI, the results of our study provide additional valuable clues for the prevention of MI. For future studies, more high-quality longitudinal and interventional studies are needed to explore the underlying mechanisms of the relationships between ambient air pollution and MI, especially in Asia and other middle- and low-income countries. Besides, it is recommended that relevant departments must adopt a comprehensive particulate matter control policy to promote health and reduce the burden of MI.

## Data Availability Statement

The original contributions presented in the study are included in the article/Supplementary Material, further inquiries can be directed to the corresponding author/s.

## Author Contributions

LZ, QZ, YZ, SC, and CL conceived and designed the study. LZ, QZ, WF, ZZ, HX, SY, JM, YZ, SC, and CL participated in the acquisition of data. LZ and QZ analyzed the data. YZ, CL, and SC gave advice on methodology. LZ drafted the manuscript. LZ, QZ, WF, ZZ, HX, SY, JM, YZ, SC, and CL revised the manuscript. All authors read and approved the final manuscript.

## Conflict of Interest

The authors declare that the research was conducted in the absence of any commercial or financial relationships that could be construed as a potential conflict of interest.
