# Supplementary material for: Long-Term Exposure to Ambient Air Pollution and Myocardial Infarction: A Systematic Review and Meta-Analysis
Source: Front Med (Lausanne). 2021 Mar 17;8:616355. doi: 10.3389/fmed.2021.616355 (PMC8010182; doi:10.3389/fmed.2021.616355)
Supplement: Supplementary file 1 [file Data_Sheet_1.PDF]

Quality Evaluation

		The selection of study population				Comparability between groups	Outcome measurement			
**Researchers**	**Time**	**Representativeness of exposure cohort**	**Choice of non-exposed cohort**	**Determination of exposure**	**No ending event at the beginning**	**Consider the comparability of the exposed group and the unexposed group when designing and statistical analysis**	**Outcome assessment**	**Is the follow-up adequate**	**Completeness of follow-up**	**Score**
Jaana Hartiala	2016	1	1	1	1	1	0	1	1	7
Laura A	2016	1	1	0	1	0	1	1	1	6
Hyeanji Kim	2017	1	1	1	0	1	1	1	1	7
Teresa To	2015	1	1	1	0	1	0	1	1	6
Silvia Koton	2013	1	1	1	1	1	1	1	1	8
Laura A	2017	1	0	1	1	1	1	0	1	6
Michael J	2011	0	1	1	1	1	1	1	1	7
Kristin A	2007	1	1	1	1	1	1	1	1	8
Richard W	2012	1	1	1	1	1	1	1	1	8
George S	2018	1	1	0	1	1	1	1	1	7
Antonella Zanobetti	2007	1	1	1	1	1	1	1	1	8
Robin C	2009	1	0	1	0	1	1	1	1	6

Robin C	2008	1	1	1	0	1	1	1	1	7
Harris He´ ritier	2018	1	1	1	1	1	1	1	1	8
Hong Chen	2016	1	1	1	1	1	1	1	0	7
C. Arden Pope	2004	1	1	1	1	1	0	1	1	7
Rob Beelen	2014	1	1	1	0	1	1	1	1	7
R Beelen	2009	1	1	1	1	1	1	1	1	8
Cathryn Tonnea	2015	1	1	1	1	1	0	1	1	7
Robin C	2011	0	1	1	1	1	0	1	1	6
Stephanie von Klot	2005	1	1	1	1	0	1	1	1	7
Anke Huss	2010	0	1	1	0	1	1	1	1	6
Ali O.Malik	2019	1	0	1	1	1	1	1	1	7
Vicki Myers	2013	1	1	0	1	1	1	0	1	6
Daniela Nuvolone	2011	1	1	1	0	1	1	1	1	7
Krishnan Bhaskaran	2011	1	1	1	1	1	1	1	1	8
Giulia Cesaroni	2014	1	1	1	1	0	1	1	1	7
